# Increasing Exercise Intensity Reduces Heterogeneity of Glucose Uptake in Human Skeletal Muscles

**DOI:** 10.1371/journal.pone.0052191

**Published:** 2012-12-20

**Authors:** Ilkka Heinonen, Sergey V. Nesterov, Jukka Kemppainen, Toshihiko Fujimoto, Juhani Knuuti, Kari K. Kalliokoski

**Affiliations:** 1 Turku PET Centre, University of Turku and Turku University Hospital, Turku, Finland; 2 Research Centre of Applied and Preventive Cardiovascular Medicine, University of Turku and Turku University Hospital, Turku, Finland; 3 Clinical Physiology and Nuclear Medicine, University of Turku and Turku University Hospital, Turku, Finland; 4 I.M. Sechenov Institute of Evolutionary Biochemistry and Physiology, Saint-Petersburg, Russia; 5 Center for the Advancement of Higher Education, Tohoku University, Sendai, Japan; University of Sydney, Australia

## Abstract

Proper muscle activation is a key feature of survival in different tasks in daily life as well as sports performance, but can be impaired in elderly and in diseases. Therefore it is also clinically important to better understand the phenomenon that can be elucidated in humans non-invasively by positron emission tomography (PET) with measurements of spatial heterogeneity of glucose uptake within and among muscles during exercise. We studied six healthy young men during 35 minutes of cycling at relative intensities of 30% (low), 55% (moderate), and 75% (high) of maximal oxygen consumption on three separate days. Glucose uptake in the quadriceps femoris muscle group (QF), the main force producing muscle group in recreational cycling, and its four individual muscles, was directly measured using PET and 18F-fluoro-deoxy-glucose. Within-muscle heterogeneity was determined by calculating the coefficient of variance (CV) of glucose uptake in PET image voxels within the muscle of interest, and among-muscles heterogeneity of glucose uptake in QF was expressed as CV of the mean glucose uptake values of its separate muscles. With increasing intensity, within-muscle heterogeneity decreased in the entire QF as well as within its all four individual parts. Among-muscles glucose uptake heterogeneity also decreased with increasing intensity. However, mean glucose uptake was consistently lower and heterogeneity higher in rectus femoris muscle that is known to consist of the highest percentage of fast twitch type II fibers, compared to the other three QF muscles. In conclusion, these results show that in addition to increased contribution of distinct muscle parts, with increases in exercise intensity there is also an enhanced recruitment of muscle fibers within all of the four heads of QF, despite established differences in muscle-part specific fiber type distributions. Glucose uptake heterogeneity may serve as a useful non-invasive tool to elucidate muscle activation in aging and diseased populations.

## Introduction

Proper muscle activation is a key feature of sports performance as well as survival in different tasks in daily life. The increase in power output is accomplished by increasing the firing frequency of motor units, or by recruiting new motor units within a muscle. Traditionally, muscle activation has been measured using electromyography (EMG) and it still remains an important tool in biomechanical research. Yet, it has its own limitations, such as it represents fairly small and localized region of the muscle, quantitation is not straightforward, and mostly isometric contractions have been used as a research model. With this technique researchers have nevertheless been able to demonstrate that activation of the muscle fibers can be affected by aging [1] and diabetes [2], but the basic physiology behind this phenomenon is still incompletely understood.

The evidence for orderly recruitment of motor units is also provided by whole body exercise performed both in animals [3] and humans [4], [5] using glycogen depletion approach. By this approach it has been reported that there is a sequential depletion of glycogen in type I (slow twitch oxidative fibers) and type II fibers (fast twitch glycolytic fibers) [3]–[5]. The interpretations of muscle fibre activations derived from this approach are however complicated by the fact that during low intensity exercise lipid oxidation predominates over energy from carbohydrates, and muscle glycogen is used mainly during high intensity exercise above lactate threshold [6]–[9]. Additionally, small muscle samples in humans also represent localized and superficial muscles such as m. vastus lateralis, whereas muscle fibre type distribution is known to differ among the different muscles [10], [11]. Although it is evident that muscles in humans are not that categorically organized to type I and type II muscles as they can be in animals, autopsy studies clearly show that for instance in m. quadriceps femoris (QF), m. vastus intermedius that is located deep within a muscle group and is in a anatomical position to resist gravity, contains the highest percentage of type I muscle fibers compared to the other three superficial muscles, especially m. rectus femoris [10], [11].

As in addition to lipid and glycogen utilization glucose derived from plasma also importantly contributes to fuel the muscles during exercise, and apparently in a very balanced way in terms of increasing exercise intensity [12], [13], we reasoned that it could also applied to investigate the activation of muscles and muscle fibers during exercise. For this purpose positron emission tomography (PET) with [18F]-fluoro-deoxy-glucose ([18F]-FDG) as a glucose tracer, is well-established approach to measure muscle metabolic activity [14]–[21]. Apart from changes in the mean metabolic activity, PET provides also measure of heterogeneity within the tissue and this feature has been used during the past two decades to measure blood flow heterogeneity in the muscles and the mechanisms responsible for its control [22]. However, studying the heterogeneity of glucose uptake instead of blood flow is of importance due to the fact that within normal physiological ranges, they are not dependent on each other [23]–[28]. Given that glucose uptake can be used to estimate muscle activation, it can also be assumed that the heterogeneity of glucose uptake provides an estimate of activation of muscle fibers within the muscles. That is, when more motor units and thus muscle fibers are increasingly activated, for instance during exercise with increasing intensities, glucose uptake heterogeneity should decrease.

Animal studies suggest that muscle fibre type distribution and thus activation patterns differ among the different muscles during exercise [29]. Therefore, we sought to elucidate in the present study whether this phenomenon also applies to humans. We hypothesized that there would be a decrease in glucose uptake heterogeneity *among* the mean values of the four heads of QF muscle group, which would indicate more uniform activation of different muscles with increasing exercise intensity. On the other hand we also hypothesized to observe decreased glucose uptake heterogeneity *within* the different muscles of QF, which could be interpreted as a recruitment of new muscle fibers within the muscles induced by enhanced recruitment of new motor units with increasing exercise intensity. As a result, we found that these assumptions were fulfilled, although mean glucose uptake was always the lowest and heterogeneity the highest in m. rectus femoris known to consist of highest percentage of type II fibers compared to the three other QF muscles.

## Methods

### Subjects

Six healthy males participated in this study (age: 31±8 years, height: 181±6 cm, weight: 80±13 kg and VO_2 max_: 49±12 ml/kg/min). The joint Ethical Committee for Turku University Central Hospital and the University of Turku approved the study protocol. The purpose and potential risks of this study were explained to the subjects, and written consents for the study were obtained. The study was conducted according to the principles expressed in the Declaration of Helsinki. The subjects were instructed to maintain their regular diet; no dietary manipulations were performed. They fasted for at least 12 h before the experiments, and physical activity was prohibited for at least 24 h before the experiments.

### Study Design

**Figure 1:**
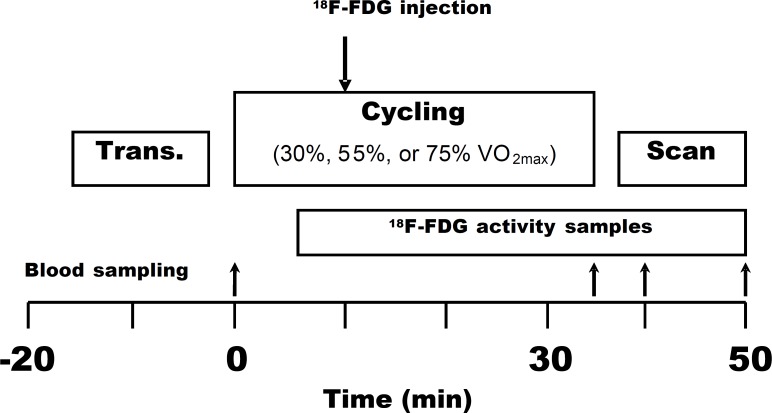
Study design. The bigger arrow indicates the moment of ^18^F-FDG injection 10 min after the beginning of exercise and the smaller arrows indicate the times of blood sampling. Trans. – transmission scanning; VO_2 max_ – maximal oxygen consumption.

The study design of the present study followed similar established protocols and principles as has been previously performed in our Centre [14], [19]. Each subject was studied on three separate days within a 3 week period; each study was performed at least 2 days apart. The study design is shown in **Figure 1**. Before the experiment, subjects rested in bed for 30 min. During that period, two Teflon catheters were inserted into the antecubital veins of a subject, one for an injection of ^18^F-FDG and the other for blood sampling of arterialized venous blood, which was obtained by heating the hand and forearm during exercise and imaging. Plasma glucose samples were drawn at the start, then at 35, 40, and 50 min. Arterialized venous blood samples (obtained by heating forearm by a warm pillow) for measurement of plasma radioactivity were obtained for each study visit separately. This is the normal practise in clinical PET studies as direct arterial line is generally considered too invasive ethically, especially in investigations with healthy subjects. Transmission scan of the femoral region was performed before the exercise, and the areas to be scanned after an exercise were carefully marked on the subject’s skin. The subject pedaled the bicycle ergometer (828E Monark, Varberg, Sweden) on separate days at the workloads of 30%, 55%, or 75% VO_2_ max at 60 rpm. After 10 min of exercise, ^18^F-FDG (156.2±2.8 MBq) was injected, and exercise was continued for another 25 min. The total exercise time was 35 min. A 12-min static PET scan of the femoral region was performed immediately, starting within 4–6 min after the 35 min of exercise.

### PET Tracer, Image Acquisition, and Processing

18F-2-fluoro-2-deoxyglucose (^18^F-FDG), an analog of glucose, was synthesized through the automated procedure using modified method of Hamacher et al. (1986). The specific radioactivity of the synthesized ^18^F-FDG was 74 GBq·µmol^−1^, and its radiochemical purity exceeded 98%. An eight-ring ECAT 931/08-tomograph (Siemens/CTI, Knoxville, TN, USA) with an axial resolution of 6.7 mm and an in-plane resolution of 6.5 mm was used. All data were corrected for dead time, decay, and measured for photon attenuation. Static FDG scans were reconstructed into a 128×128 matrix using a 2D-ordered subsets expectation maximization and median root prior (2D OSEM-MRP) reconstruction with 150 iterations and the Bayesian coefficient 0.3. The final in-plane resolution of the reconstructed images was 8 mm.

Once administered to the body, ^18^F-FDG is transported into cells via the glucose transporter protein GLUT4 and then phosphorylated to FDG-6- phosphate in the presence of hexokinase or glucokinase. It is metabolically trapped in cells without further progression of the glycolytic pathway because FDG-6- phosphate is a very poor substrate for glucose-6-phosphate isomerase for further glycolysis. Therefore, the amount of trapped FDG-6-phosphate inside the tissue reflects the level of glucose utilization in the applied study condition. This trapping nature is useful for quantification of glucose metabolism, and [18F]FDG also allows for imaging muscular or inner organ glucose metabolism without any restriction of body movements during an exercise as the PET scanning can be performed after the cessation of exercise.

### Regions of Interests

**Figure 2:**
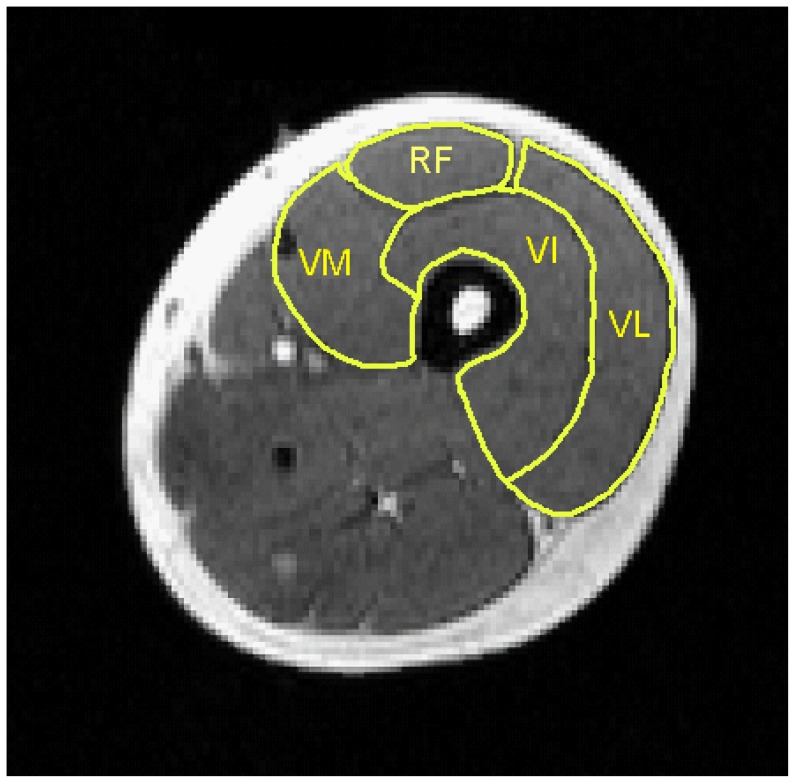
Regions of interest (ROI) drawn on MRI image example over the muscles constituting the quadriceps muscle. RF – m. rectus femoris, VL – m. vastus lateralis, VM – m. vastus medialis, VI – m. vastus intermedius.

Regions of interest (ROI) were drawn on four adjacent planes in the middle of a thigh between the patella and the anterior superior iliac spine similarly in each exercise intensity, both in the right and the left quadriceps muscles (*musculus quadriceps femoris* – QF). The ROIs were drawn for QF and all the constituent muscle of the quadriceps group: rectus femoris (RF), vastus intermedius (VI), vastus lateralis (VL), and vastus medialis (VM) **(** **Figure 2** **)**. The average glucose uptake values of these ROI from both legs were used for calculations.

### Calculation of Regional Glucose Uptake

Quantification of GU was based on the method developed by Sokoloff et al. [30] and similar principles that are frequently applied in our Centre in various experiments where imaging of GU takes place after short-term physiological intervention, such as after cold exposure when GU of brown adipose tissue is measured [31], [32]. The glucose uptake rate (rGU) was obtained by multiplying the fractional rate of tracer uptake Ki by the plasma glucose concentration [Glc]p divided by a lumped constant term (LC): *rGU = [Glc]p/LC) × Ki.* The lumped constant accounts for differences in the transport and phosphorylation of ^18^F-FDG and glucose. A lumped constant value of 1.2 was used for skeletal muscle [33]. The *Ki* was calculated as *Cm/Cp: ∫Cp/Cp*, where *Cm* is skeletal muscle radioactivity, *Cp* is plasma radioactivity concentration, and *∫Cp* is the integral of plasma radioactivity concentration from the time of injection to the end of the scan.

### VO_2 max_ Measurement

VO_2 max_ was determined using direct measurement of the oxygen consumption rate and a bicycle ergometer (model 800 S, Ergoline, Mijnhardt, The Netherlands) with a continuous incremental protocol with 30 watt increments every two minutes starting from 50 watt until exhaustion.

### Other Measurements

Plasma glucose was determined by a glucose oxidase method (GM7 Analyser, Analox Instruments, Hammersmith, London, UK).

### Statistical Analysis

Statistical analysis was performed using SAS/STAT statistical software (version 8.2, SAS institute Inc., Cary, NC, USA). For multiple comparisons, ANOVA followed by the Tukey post-hoc test for the analysis of differences between muscles and exercise intensities was applied. *P* values <0.05 were considered statistically significant. The results are expressed as mean ± SD.

## Results

The absolute cycling intensities representing workloads of 30%, 55%, or 75% VO_2_ max were 93±28, 183±37, and 229±35 watts. Lactate concentrations were before the exercise 1.0±0.4, 0.9±0.3, and 1.2±0.3 mmol/L and at the end of each bout of exercise 1.1±0.3, 3.9±1.4, 9.4±2.6 mmol/L respectively.

**Figure 3:**
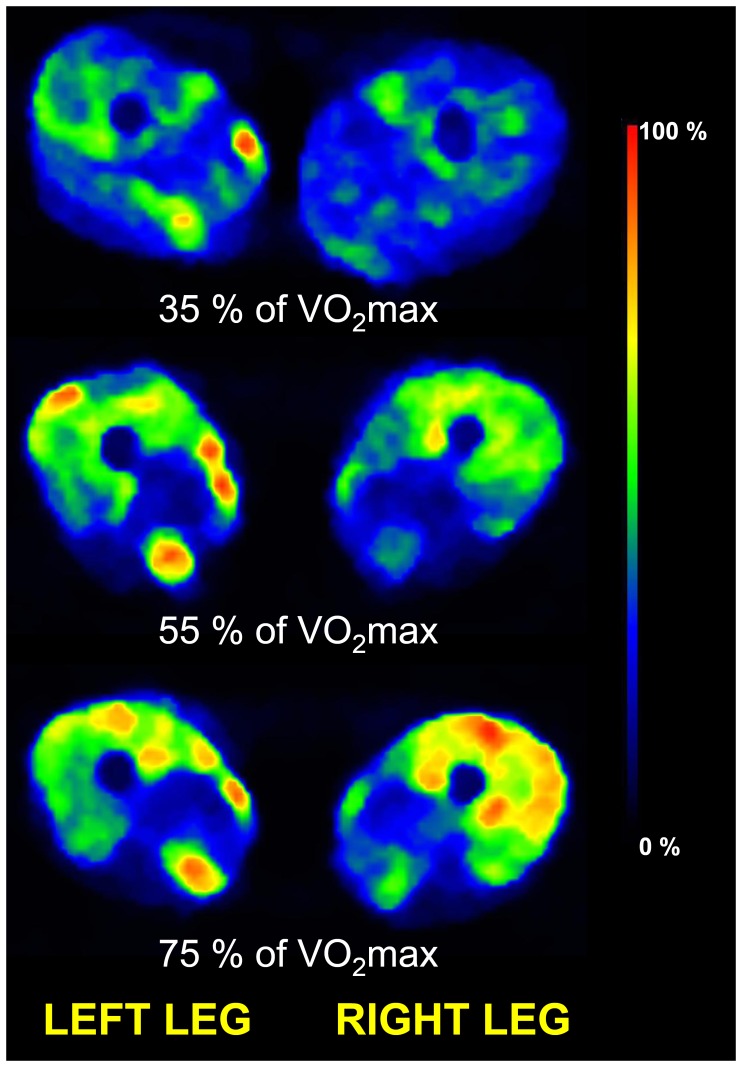
Representative cross-sectional PET images of glucose uptake of the thigh region during steady-state cycling with three different exercise intensities in one of the subjects. The color scale shows the glucose uptake in a relative scale.

**Figure 4:**
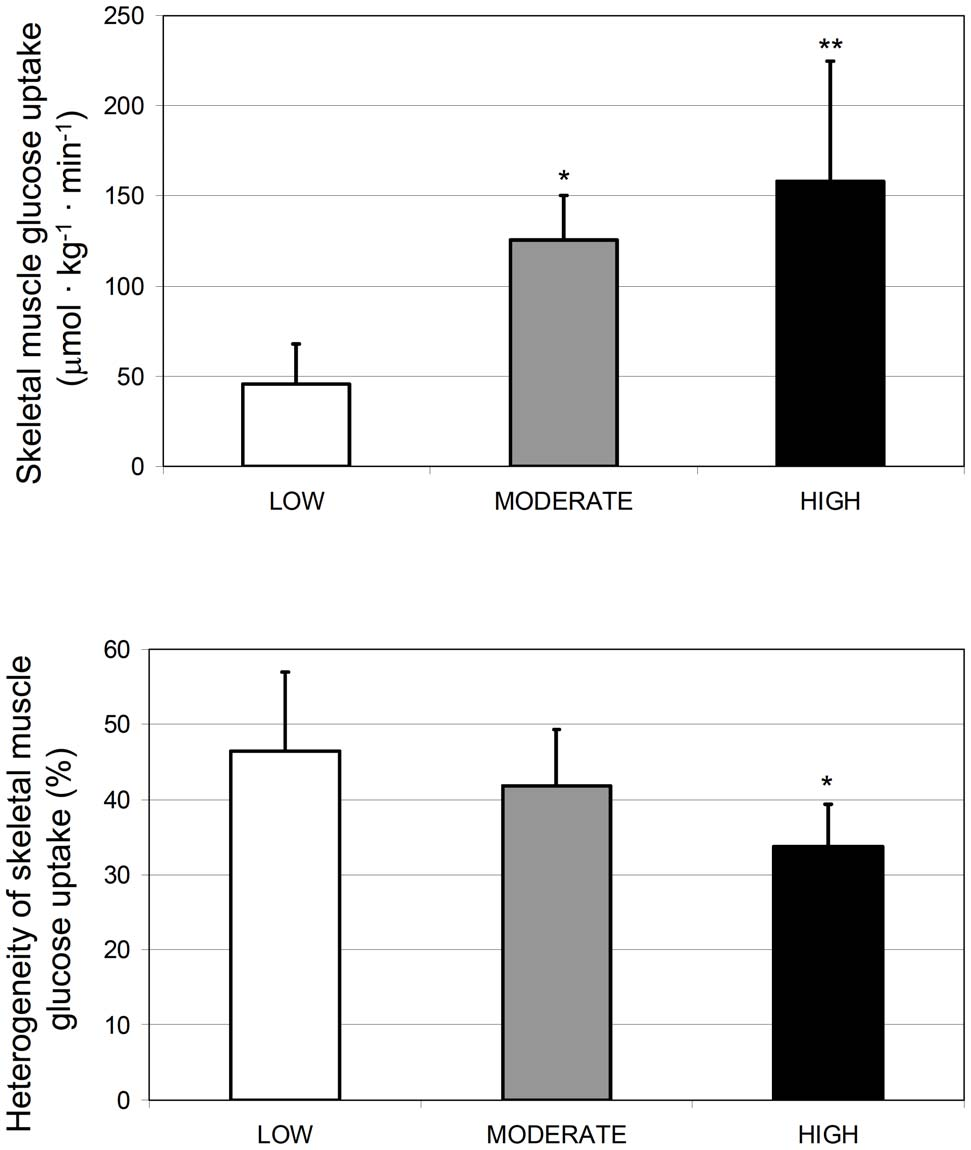
Glucose uptake during exercise at different exercise intensities. Mean glucose uptake and its heterogeneity in the whole quadriceps femoris muscle group are shown in panels A and B, respectively. *p<0.05 and **p<0.01 compared to LOW.

**Figure 5:**
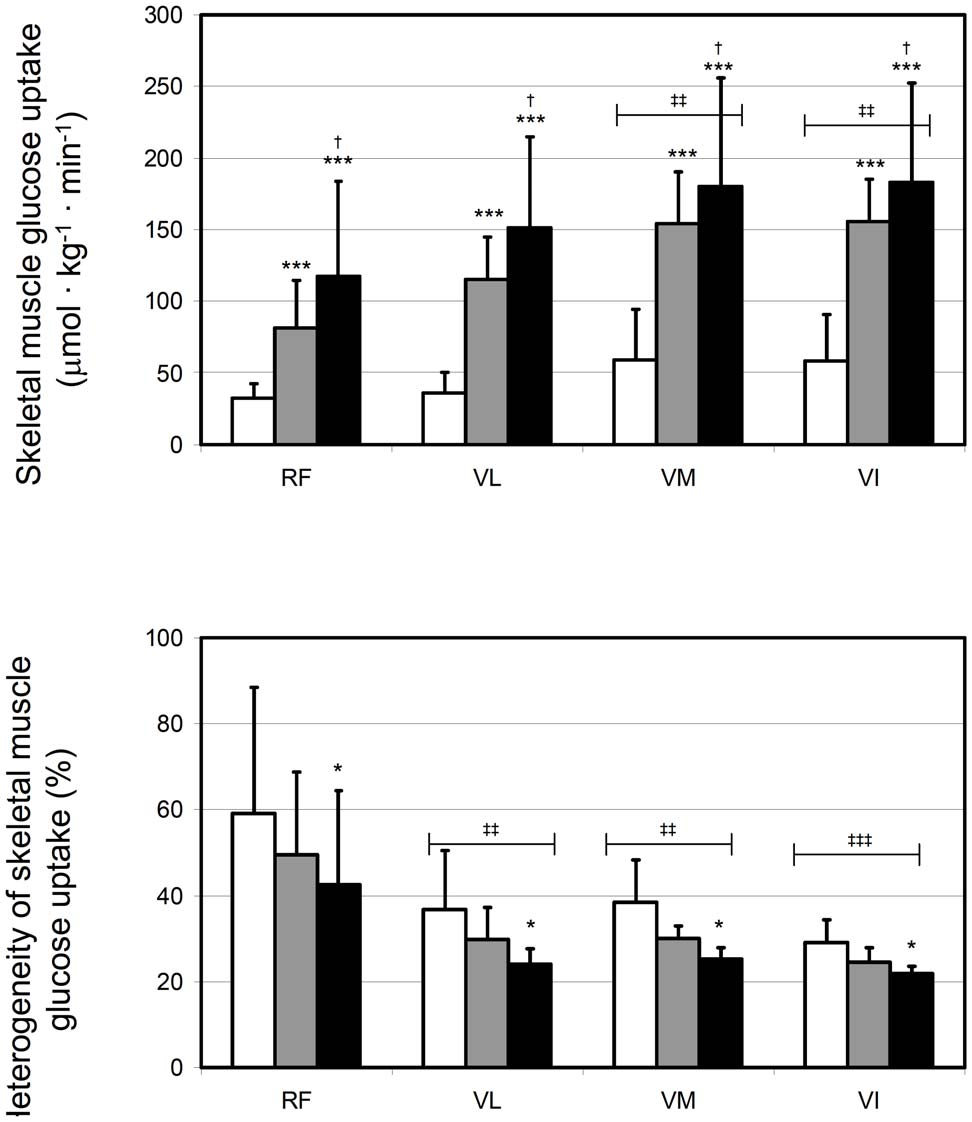
Glucose uptake during exercise in different muscles of quadriceps femoris at different exercise intensities. Mean glucose uptake and its heterogeneity in the four individual muscles are shown in panels A and B, respectively. RF – m. rectus femoris, VL – m. vastus lateralis, VM – m. vastus medialis, VI – m. vastus intermedius. No interaction between intensity and muscle was found in ANOVA, but both main effects (intensity and muscle) were significant and the following pair-wise differences were found: *p<0.05 and ***p<0.001 compared to LOW intensity, †p<0.05 compared to MODERATE intensity, and ‡‡p<0.01 and ‡‡‡p<0.001 compared to RF muscle. White, grey and black bars represent LOW, MODERATE and HIGH exercise intensities, respectively.

The representative glucose uptake in QF muscles is illustrated in Figure 3. Quantitatively, mean glucose uptake in the entire QF increased significantly from low to moderate (p = 0.001), but not anymore significantly from moderate to high exercise intensity (p = 0.41) (Figure 4A). The responses in each four parts of QF followed the same pattern, although the increase from low to moderate was almost significant (p = 0.057, Figure 5A). Glucose uptake heterogeneity within the whole QF decreased with increase in exercise intensity (Figure 4B). This was partly due to decrease in heterogeneity among the mean values of four heads of QF along with increasing intensity (35±13, 30±10, and 22±9%, p = 0.017). On the other hand, glucose uptake heterogeneity decreased significantly also within the four heads of QF from lowest to highest intensity (p = 0.016, Figure 5B). Mean glucose uptake was however always higher in VI, VM and glucose uptake heterogeneity lower in VI, VM and VL compared to RF (Figure 5A and 5B, respectively).

## Discussion

We investigated the effects of different exercise intensities on quadriceps femoris (QF) muscle glucose uptake heterogeneity in humans during cycling. We found a decrease in QF muscle glucose uptake heterogeneity when exercise intensity increased. This stemmed from more uniform activation of the four muscles of the QF, but also from decreased glucose uptake heterogeneity within the individual muscle parts. These results suggest that with increase in exercise intensity there is an increased contribution of those muscles which are fairly inactive during low intensity, but also that there is an enhanced recruitment of motor units and ultimately muscle fibers within all of the four heads of QF. We also found that there was lower mean glucose uptake and higher glucose uptake heterogeneity in m. rectus femoris compared to especially m. vastus intermedius and m. vastus medialis. This is most likely due to lower oxidative capacity, capillarity, and GLUT4 content of rectus femoris as it has been shown to constitute usually mostly of type II muscle fibers that possess all these features [10], [11] [34]–[39], although it cannot be excluded that biomechanical activation of this muscle simply is low in cycling.

The increase of glucose uptake in skeletal muscle during exercise involves acceleration of two main processes: increased supply of glucose via the increased capillary perfusion and increased membrane transport via the translocation of GLUT4 from a designated intracellular storage compartment to the plasma membrane and transverse tubules [40]. The mean glucose uptake in entire QF as well as in its four separate parts increased from low to moderate intensity, but not anymore significantly from moderate to highest intensity. Yet, the energy production in the QF muscle must have increased as it performed the exercise at much higher intensity. It is highly likely that endogenous glucose from glycogen was increasingly responsible to be burned as energy during the highest exercise intensity as it is more efficient to take energy from glycogen compared to extracellular glucose utilization. Moreover, it is also likely that lactate was also used for energy in skeletal muscle during high intensity exercise as previously suggested for heart and brain [41], [42]. Interestingly, it was found that despite of the fact that mean glucose uptake tended to level off towards the highest exercise intensity, the heterogeneity of glucose uptake still decreased concomitantly.

Based mostly on one-leg exercise model, it is well known that considerable heterogeneity between and within muscles is observed in blood flow and glucose uptake both at rest and during exercise [25], [43]–[48]. This was the case also during whole body exercise in the present study as we found that glucose uptake was lower in RF than in VM and VI at all exercise intensities. Additionally, within-muscle glucose uptake heterogeneity was always higher in RF compared to VL, VM and VI. This result is intriguing, as RF type II fibers usually represent ∼65% of all fibers, whereas the percentages for VL, VM and VI are 55–60%, 45–50% and 45–50%, respectively [10], [11]. Moreover, there is also evidence that glucose uptake goes along with the amount of protein GLUT4, which varies according to the fiber type distribution within muscles [34], [35], [39]. Hence, although it cannot be excluded that during cycling RF simply is not activated to that extent than the other three parts of QF, it is likely that differences in fiber type distribution, most notably between RF and VM and VI, account for the observed difference in mean glucose uptake and its heterogeneity during exercise. It is also likely that the remaining heterogeneity is explained by the fact that muscle activation and thus high forces cannot be reached in cycling, especially by untrained subjects. However, it is likely that lower heterogeneity values could be obtained by highly trained subjects such as highly trained cyclists, who could obtain and sustain much higher pedal forces in cycling.

When the results of the present study are integrated with the evidence that has accumulated during the past several decades dealing with the activation of muscle fibers during exercise, it can be concluded that they are well in accordance with both animal [29] and human [4], [5] data. When in particular animal data is considered, it appears evident that muscles rich in type I fibers always have the highest metabolic activity during exercise, regardless of the exercise intensity [29], [49]. An exception to animal studies however is that in humans all muscles are activated relatively to a similar extent with increasing exercise intensity, but in animals the muscles consisting mostly of type II fibers show the largest relative increase in activation [29], [49]. This may be due to the fact that fiber type distribution is not so distinctly different between muscles in humans than it is in animals [10], [11].

The results of the present study also strengthen the interpretations drawn from both EMG [50], [51] and glycogen depletion studies [3]–[5], by confirming the more uniform muscle fiber recruitment as exercise intensity increases. On the other hand our present results also extend them in several ways. Most importantly our study penetrated to the deepest regions of the muscles and comprehensively involved several muscle parts that were evaluated simultaneously. We also confirmed that conclusions drawn from glycogen depletion studies appear to apply also when glucose derived from plasma is used as a metabolic indicator for muscle fiber recruitment. Finally, the results of the present study also directly extend the previous one leg knee extensor exercise blood flow work of us [47] and that of others [52], confirming that the physiological decrease in heterogeneity with increasing exercise intensity is on one hand explained by more uniform activation of the four muscles of the QF [47], but also that increased power output is obtained mostly by recruiting more fibers rather than increasing metabolism of the fibers that are already engaged in exercise [52]. On the other hand it must also be mentioned that glucose uptake does not depend on blood flow when they are in normal physiological ranges. For instance, Bradley and colleagues documented a decrease in glucose uptake but no change in bulk blood flow during inhibition of nitric oxide synthase (NOS) during cycling exercise in healthy young men [26]. In contrary, very localised NOS blockade (infusion via microdialysis into the muscle) can lower blood flow during exercise without changing glucose uptake [27]. Moreover, there is also other evidence both from human [23]–[25] and animal studies [28] that indeed regional blood flow is not a determinant of glucose uptake within a same region. The strength of ^18^F-FDG, the glucose uptake tracer used in the present study, is that it is transported into the cells almost in the same manner as normal glucose using glucose transporters. The difference in the transport rate of ^18^F-FDG and glucose is corrected by the use of tissue specific lumped constant values which has been shown to be 1.2 for the skeletal muscle [33]. Due to very high sensitivity of PET method, the amount of ^18^F-FDG injected is negligible when compared to the amount of glucose in the blood and therefore, it does not have any effect on normal glucose uptake process or in feedback to GLUT4, and glucose uptake can be analysed tissue- and region specifically having the possibility to provide detailed information about skeletal muscle activation. Due to these encouraging results, we suggest that this glucose uptake heterogeneity approach might be valuable non-invasive tool for the investigations of altered muscle fiber activations induced by aging [1] and diabetes [2].

In conclusion, our results indicate that the decrease in glucose uptake heterogeneity with increasing exercise intensity in cycling stems from more uniform activation between the four muscles of the QF, but also from decreased glucose uptake heterogeneity within the muscles. These results suggest that in addition to increased contribution of muscle parts that are fairly inactive during low intensity, with increases in exercise intensity there is also an enhanced recruitment of motor units and thus muscle fibers within all of the four heads of QF, despite of established differences in muscle-part specific fiber type distributions. Finally, the lower mean glucose uptake and higher glucose uptake heterogeneity in m. rectus femoris compared to especially m. vastus intermedius and m. vastus medialis is likely due to lower oxidative capacity, capillarity, and GLUT4 content of rectus femoris, or simply due to the fact that biomechanical activation of this muscle is low in cycling exercise.

## Acknowledgments

The study was conducted within the Centre of Excellence in Molecular Imaging in Cardiovascular and Metabolic Research - supported by the Academy of Finland, University of Turku, Turku University Hospital and Abo Academy. The authors want to thank the contribution of the personnel of the Turku PET Centre for their excellent assistance during the study, and the subjects who participated.
